# Ischemic Preconditioning and Exercise Performance: An Ergogenic Aid for Whom?

**DOI:** 10.3389/fphys.2018.01874

**Published:** 2018-12-21

**Authors:** Moacir Marocolo, François Billaut, Gustavo R. da Mota

**Affiliations:** ^1^Physiology and Human Performance Research Group, Department of Physiology, Federal University of Juiz de Fora, Juiz de Fora, Brazil; ^2^Department of Kinesiology, Laval University, Quebec, QC, Canada; ^3^Human Performance and Sports Research Group, Department of Sport Sciences, Institute of Health Sciences, Federal University of Triangulo Mineiro (UFTM), Uberaba, Brazil

**Keywords:** sports, athletes, blood flow occlusion, enhancement, conditioning

## Introduction

Maneuvers of occlusion and reperfusion of the muscle blood flow aiming at improving exercise performance have been used since the 1950s with conflicting conclusions (Marocolo et al., 2016a). In the 80's decade, ischemic preconditioning (IPC) was defined as an intervention that consists of brief events of ischemia followed by reperfusion (Murry et al., 1986). It was described that a tissue, previously submitted to ischemic conditions, becomes more resistant to ischemia and its potential deleterious effects (Kocman et al., 2015). This remarkable clinical effect of IPC attracted sports scientists, and from 2000s many studies investigating the potency of IPC for enhancing exercise performance appeared massively in the scientific literature. Sport scientists attempted to demonstrate the beneficial effects of IPC on swimming (Marocolo et al., 2015; Ferreira et al., 2016), running (Sabino-Carvalho et al., 2017), cycling (Paradis-Deschênes et al., 2016), resistance (Marocolo et al., 2016b,c), and intermittent exercises (Marocolo et al., 2017) or general sports modalities (Incognito et al., 2016; Richard and Billaut, 2018). Nowadays, IPC is still studied for its ergogenic properties because it is simple, non-invasive, affordable and, thereby, readily applicable to exercise performance settings. While IPC can improve exercise performance, especially in endurance events, the mechanisms underlying its effects are unclear, as well as the robustness of the findings. Here we raise some methodological concerns about protocol design, data analysis, and interpretation, and discuss relevant positive effects and future directions for investigation.

## Methodological Concerns

Although some studies have reported about positive effects of IPC on physiological responses and performance (see last section below), the reader must be aware that the scientific literature does not unanimously report beneficial effects. Rather, some papers reported null (Marocolo et al., 2017) or even negative effects (Paixao et al., 2014), and to date, the positive effects are highly contentious (Marocolo et al., 2016a). The below sections present some methodological aspects that must be addressed to move this field forward and find the optimal dose of IPC, if any.

## Data Analyzes and Fitness of The Subjects

Most studies investigating the effects of IPC on exercise performance have tested recreational/amateur subjects, with limited transfer to a higher level of competition in which ergogenic aids are highly relevant. Furthermore, most studies have carried out open-looped laboratory tests of unknown duration for the participant, and there is a paucity of studies dealing with self-paced exercise (e.g., field tests). Field tests are more similar to what athletes actually experience in training/competitions than in the laboratory. So, to move this research field forward, it is now critical to focus on the potential ergogenic effects of IPC on high-level fitness athletes tested in “real world scenario.” The data analyzes of studies evaluating the efficacy of IPC are mainly based on statistical tests (*p*-value) or effect size (ES) comparisons to conclude about the presence or absence of “beneficial effects.” Although there is a lively debate about the proper statistical approach that should be considered for exercise performance studies (Batterham and Hopkins, 2015; Welsh Knight and Knight, 2015), which is beyond the topic of this opinion article, the statistical results from approaches such as *t*-tests and ANOVA or ES, should be specifically interpreted. For example, a small ES found in elite athletes may be relevant, but a moderate/large ES in recreational subjects may not be. Therefore, until more robust and clear evidence demonstrates a beneficial effect in high-level athletes with at least a small ES, it is not advised to extrapolate the results observed in lower-end fitness subjects to high-level competitors. Analyzing the fitness level of subjects among almost 50 experimental studies that measured the effects of IPC on exercise performance in healthy subjects, one of them tested elite speed skaters (Richard and Billaut, 2018) while another evaluated elite cyclists (Paradis-Deschênes et al., 2018). Subjects evaluated in all other studies were amateur or recreational athletes, or even just healthy or sedentary (non-published data), even when the title or some part of the manuscript quoted “highly trained.” In this sense, an interesting study (Foster et al., 2014) evaluating 12.8 km time trial in altitude found that running was faster when IPC was prior applied. However, their results were not statistical different and the ES was trivial (Marocolo et al., 2016a). Furthermore, another study testing swimmers (Jean-St-Michel et al., 2011) found benefits after IPC intervention, but again with trivial ES. Although the authors called “high-trained,” actually they were not, since their 100 m swimming time of about 60 s are comparable to amateur swimmer. Generally, IPC effects on field and self-paced exercises present small magnitude for being considered effective. Therefore, we strongly encourage more research in real field performance targeting truly elite individuals.

## IPC Protocols and the Potential Placebo Effect

Another issue that may have prevented scientists from reaching a consensus to date is the substantial variation among the IPC protocols (1–4 cycles of 2–5 min of ischemia) applied in exercise performance studies. While the potency of IPC may exist, it is possible that decades of research have not yet found the “correct protocol.” While no effect on exercise performance have been demonstrated in several studies using standard protocols (e.g., 3 or 4 × 5-min occlusion/reperfusion; total ischemia of 15–20-min) (Marocolo et al., 2016a), a clinical study has shown that only 4 min of occlusion may be sufficient to reach a threshold for ischemic stimulus, regardless of the number of ischemic cycles (Ghosh et al., 2000). Since more ischemia cycles have not promoted greater enhancements in exercise performance (Cocking et al., 2018), new investigations should test the efficacy of shorter protocols. Intriguingly, shorter IPC protocols have not been examined, and if proven beneficial, these might offer a better option for athletes and coach staff due to obvious logistical reasons.

The placebo and/or nocebo effects are both methodological confounding factors in studies involving any potential ergogenic aid (Marocolo et al., 2015, 2017; Sabino-Carvalho et al., 2017). Specifically with IPC, a scientific debate raised questions about the real efficacy of IPC on performance. For instance, only 24% of the studies included in a systematic review, found beneficial effects of IPC on performance (or physiological variables) when a placebo control group was present (da Mota and Marocolo, 2016; Incognito et al., 2016). Vice versa, when a placebo group was not included in the analysis, the prevalence for positive effects increased (Incognito et al., 2016) (da Mota and Marocolo, 2016). Additionally, several studies found similar positive effects between low and high-pressure cuffing (i.e., SHAM and IPC) (Marocolo et al., 2016a,c; Sabino-Carvalho et al., 2017; Thompson et al., 2018). Thus, we could speculate that beyond potential placebo effects (Marocolo et al., 2015; Sabino-Carvalho et al., 2017), a bidirectional brain-body integration mechanism may promote physiological responses through mechanical-sensory receptors (Taylor et al., 2010; Cromwell and Panksepp, 2011).

Furthermore, it might be possible that the beneficial effect of IPC includes a lower perception of fatigue as a potential mechanism. Indeed prior experiments have been suggested that IPC can potentially desensitize group III and IV nerve endings to metabolite accumulation. It is stated that type III and IV nerve endings exert contribution to the cardiovascular regulation during exercise (Amann et al., 2011), which could direct contribute to changes in performance. These types of nerve (III and IV) modulate the sympathetic tone based on mechanical and metabolic conditions on the working muscle, acting type III mainly as mechano- and IV as metabo-receptors (Nobrega et al., 2014). When the end part of this nerve is stimulated, increases in sympathetic tone, regulating hemodynamic parameters such as heart rate and cardiac contractility (Crisafulli et al., 2011; Marongiu et al., 2013).

It was found increases in handgrip performance after IPC, without changes in blood flow, conductance and muscular deoxygenation, with differences in the slowing of contraction and relaxation throughout the exercise (Barbosa et al., 2015). This finding suggests that IPC could affect some neural pathway. Corroborating their results, another study (Mulliri et al., 2016) investigated the effect of IPC on the hemodynamics during metaboreflex recruitment and found reductions in mean arterial pressure response and impairs venous return, possible through increases in nitric oxide production. Although the second study did not support the hypothesis that IPC improves performance in exercise with limited muscle mass, they showed that IPC affects hemodynamics. Future research should better clarify the relation between IPC and the role of type III and IV nerve endings.

## Positive Effects of IPC

This opinion paper has presented current scientific pitfalls to data interpretation about the efficacy and applicability of IPC. Nonetheless, we recognized that IPC has been shown to elicit beneficial, ergogenic adaptations conducive to enhanced physical capacity. The recent meta-analysis performed by Salvador et al. (2016) demonstrated a real impact on sports performances. When all types of performances were combined, IPC yielded a “small” beneficial (ES 0.43) effect with no chance of observing a negative impact (ES range 0.28–0.51). The most robust beneficial impact was reported for “aerobic” (>90 sec) performances with a “moderate” (ES 0.51) impact. Results for performances of shorter duration were not convincing (Marocolo et al., 2016b; Salvador et al., 2016). A detailed look at the scientific literature reveals relevant findings for athletes. For example, aerobic power output measured during an incremental cycle test to volitional fatigue went up by 3% (p<0.01) from 366W to 372W in athletes (de Groot et al., 2010). This finding is in line with performances of runners reported running a 5-km time trial 34 s faster (p<0.05) after using IPC (Bailey et al., 2012). IPC has been reported to improve maximal performance in various exercise modes when the oxidative system is fully taxed (de Groot et al., 2010; Bailey et al., 2012; Kjeld et al., 2014). Along this line, some evidence exists showing that IPC can, in some cases, enhance performance during the hypoxic insult. A study reported greater power output and faster time to complete a 5-km time trial in cyclists at 2,500-m simulated altitude (Paradis-Deschênes et al., 2018). These enhanced aerobic performances may be related to acute molecular and vascular adaptations that promote local vasodilation, enhance blood flow, and ultimately improve O_2_ delivery and utilization (Tapuria et al., 2008; Beaven et al., 2012). However, trends in muscle oxygenation are not always clear after IPC. Studies have reported attenuated (Kido et al., 2015; Patterson et al., 2015), accentuated (Barbosa et al., 2015; Paradis-Deschênes et al., 2016) and accelerated dynamics (Kido et al., 2015; Tanaka et al., 2016), which complicates the understanding of the IPC-induced physiological mechanisms.

Although meta-analyses do not favor IPC in enhancing shorter performances see Salvador et al., 2016, some studies still reported interesting findings for the “anaerobic” athlete. The muscular force developed during repeated maximal isokinetic contractions was enhanced in strength-trained athletes (Paradis-Deschênes et al., 2016) and swimmers could produce the fastest times during 50-m (1.2%, *p* < 0.05, Lisbôa et al., 2017) and 100-m (1.1%, *p* < 0.01, Jean-St-Michel et al., 2011) sprints after using IPC compared to sham occlusions. These data are certainly of practical importance during competitions. However, other studies could not report any superior performances after IPC (Patterson et al., 2015), which demonstrates that the context of the application, the protocol and, probably, the type of athletes influence the outcomes of this technique.

## Future Directions and Conclusions

This opinion piece is aimed at raising awareness in athletes and coaches, and to call upon researchers to urgently address current experimental pitfalls that obscure our understanding. We believe that future studies should test shorter protocols (e.g., 2 × 2–3 min occlusion/reperfusion), which are more time-efficient (e.g., 8–12 min vs. 40 min) and more easily inserted in real-world settings of athletes/competitions if positive and meaningful findings are confirmed. Also, testing treatments controlled by different cuffing pressures (i.e., SHAM, IPC, and no cuff—control) should assess the effect of IPC on higher fitness subjects (i.e., elite athletes). Then only, we may be able to draw robust conclusions as to whether IPC is suitable for recreational practitioners and/or elite athletes.

## Author Contributions

MM and GdM made substantial contributions to the conception, design, and drafting of the work, as well as the analysis and interpretation of data for the work. MM, FB, and GdM revised it critically for important intellectual content. MM, FB, and Gdm provided approval for publication of the content. MM, FB, and GdM agreed to be accountable for all aspects of the work in ensuring that questions related to the accuracy or integrity of any part of the work were appropriately investigated and resolved.

### Conflict of Interest Statement

The authors declare that the research was conducted in the absence of any commercial or financial relationships that could be construed as a potential conflict of interest.
